# Brief Exercise Increases Peripheral Blood NK Cell Counts without Immediate Functional Changes, but Impairs their Responses to *ex vivo* Stimulation

**DOI:** 10.3389/fimmu.2013.00125

**Published:** 2013-05-29

**Authors:** Anne-Laure Millard, Piero V. Valli, Georg Stussi, Nicolas J. Mueller, Gisella Puga Yung, Jörg D. Seebach

**Affiliations:** ^1^Division of Infectious Diseases and Hospital Epidemiology, University Hospital, Zürich, Switzerland; ^2^Laboratory for Transplantation Immunology, University Hospital, Zürich, Switzerland; ^3^Clinic for Haematology, University Hospital, Zürich, Switzerland; ^4^Laboratory for Transplantation Immunology, University Hospital and Medical Faculty Geneva, Geneva, Switzerland

**Keywords:** NK cells, cytotoxicity, exercise, IFNγ production, CD56^bright^

## Abstract

Physical as well as psychological stress increases the number of circulating peripheral blood NK cells. Whereas some studies found a positive correlation between exercise and NK cell counts and cytotoxic activity, others showed that, for example, heavy training leads to a decrease in per cell NK cytotoxicity. Thus, the impact of exercise on NK cell function and eventually on altered immunocompetence remains to be elucidated. Here, we investigated whether a single bout of brief exercise, consisting in running up and down 150 stair-steps, affects the number and function of circulating NK cells. NK cells, obtained from 29 healthy donors, before and immediately after brief exercise, were assessed for numbers, phenotype, IFNγ production, degranulation, cytotoxicity, and *in vitro* response to stimulation with IL-2, IL-2/IL-12, or TLR2 agonists. Running resulted in a sixfold increase in the number of CD3^−^/CD56^+^ NK cells, but decreased the frequency of CD56^bright^ NK cells about twofold. Brief exercise did not significantly interfere with baseline IFNγ secretion or NK cell cytotoxicity. *In vitro* stimulation with IL-2 and TLR2 agonists (lipoteichoic acid, and synthetic triacylated lipopeptide Pam3CSK4) enhanced IFNγ-secretion, degranulation, and cytotoxicity mediated by NK cells isolated pre-exercise, but had less effect on NK cells isolated following exercise. There were no differences in response to combined IL-2/IL-12 stimulation. In conclusion, having no obvious impact on baseline NK functions, brief exercise might be used as a simple method to significantly increase the number of CD56^dim^ NK cell available for *in vitro* experiments. Nevertheless, the observed impaired responses to stimulation suggest an alteration of NK cell-mediated immunity by brief exercise which is at least in part explained by a concomitant decrease of the circulating CD56^bright^ NK cell fraction.

## Introduction

Being part of the first line of host defense against infections and malignant diseases, natural killer (NK) cells represent an important cell population of the innate immune system (Caligiuri, 2008; Vivier et al., 2011). Characterized by spontaneous “natural” cytotoxicity, NK cells also provide an early source of immunoregulatory cytokines thus being able to influence adaptive immune responses. Representing about 5–15% of peripheral blood lymphocytes, the majority of human NK cells (≅90%) are distinguished by low density-expression of CD56 (CD56^dim^) and high expression of CD16, whereas the remaining NK cells are CD56^bright^ CD16^dim/neg^ (Cooper et al., 2001). Earlier studies revealed that distinct functions can be assigned to these two NK cell subsets (Di Santo, 2008). While CD56^dim^ NK cells play a key role in natural and antibody-dependent cell-mediated cytotoxicity, the CD56^bright^ NK population is a potent producer of cytokines such as IFNγ and exhibits lower cytotoxicity (Moretta et al., 2008).

The function of NK cells is regulated by a balance of activating and inhibitory receptors as well as by several cytokines. Activating NK receptors include the natural cytotoxicity receptors (NCR: NKp30, NKp44 and NKp46), NKG2D and DNAM-1 (CD226) (for review see Brusilovsky et al., 2012). The activation-associated receptors CD25 and CD69 are related respectively to the proliferative and cytotoxic potential of NK cells (Clausen et al., 2003) whereas CD107a, the lysosome-associated membrane protein-1 (LAMP-1), has been identified as a sensitive marker for NK degranulation and cytotoxicity (Alter et al., 2004). Inhibitory receptors include the killer-cell immunoglobulin-like receptor (KIR) family and the CD94/NKG2A heterodimer which all regulate the killing function by interacting with MHC class I molecules. Finally, several pattern-recognition receptors of the TLR family are expressed by NK cells, and TLR2 (CD282) signaling was reported to directly activate NK cells *in vitro* (Becker et al., 2003; Millard et al., 2010) although this issue remains controversial due to the potential influence of contaminating myeloid cell subsets (Costantini et al., 2009).

Among other cytokines such as IL-15 and IL-18, IL-2, and IL-12 play a crucial role in NK cell proliferation, differentiation, and cytokine secretion; both NK cytotoxicity and IFNγ production are enhanced by IL-2 and IL-12 *in vitro* (Fehniger et al., 2003). All naïve NK cells constitutively express functional intermediate-affinity heterodimeric IL-2 receptor complexes (IL-2Rβγ), containing the β-chain CD122, whereas CD56^bright^ NK cells express in addition high-affinity heterotrimeric IL-2Rαβγ complexes, containing the α-chain CD25 (Nagler et al., 1990).

A pilot study on the effect of 5 min running revealed an increase of NK cell frequencies in the peripheral blood of human donors following exercise (Edwards et al., 1984). These findings have since been corroborated by numerous studies showing that physical exercise of various strength and duration lead to an increase in the circulating numbers of human NK cells (for review see Brolinson and Elliott, 2007). However, the impact of exercise on the immune function remains a matter of debate (Walsh et al., 2011). Whereas some studies found a positive correlation between exercise and NK cell counts and cytotoxicity (Nieman, 2000), others showed that, for example, heavy training was related to a decrease in per cell NK cytotoxicity (Suzui et al., 2004).

Being a major component of the innate immune system, NK exert a pivotal role in the early response to viral infections and tumors, but also in the context of solid organ transplantation and during inflammation (Caligiuri, 2008). However, several aspects of NK cell immunobiology remain to be unveiled. Having a simple method to increase the yield of human NK cells from a defined blood volume will be especially advantageous for subsequent *in vitro* studies.

In this report, we evaluated whether a single bout of brief exercise, consisting in running up and down 150 stair-steps, could be used as a simple method to increase the number of peripheral blood NK cells available for *in vitro* experiments. Therefore, we evaluated the exact magnitude of the increase in human NK cell numbers in response to brief exercise, as well as the effect on NK subset distribution and function. Overall, a sixfold increase of the number of isolated NK cells was observed, with no major impact on neither their baseline activation status, IFNγ-secretion, and cytotoxicity nor on their response to combined IL-2/IL-12 stimulation. Nevertheless, exercise impaired the *in vitro* responses to IL-2 and TLR2 agonists and preferentially released CD56^dim^ NK cells.

## Materials and Methods

### Subject characteristics and exercise

Following written informed consent, 29 healthy volunteers accustomed to physical exercise (19 males and 10 females, age range of 25–45 years,) participated in the study. Volunteers were covered by the institutional insurance, were evaluated by a study-independent institutional doctor as to being in good health, and asked whether they were willing to donate blood to be used in clinical and experimental trials. The review board of the University Hospital Zurich specifically waived the need for a formal ethical committee approval for this study. Blood samples were taken by peripheral venous puncture pre-exercise and immediately (within 5 min) post-exercise, consisting in running up and down of 150 stair-steps. Exercise lasted for 68.8 ± 14.2 s with a subsequent heart rate between 120 and 150/min.

### Cell purification and isolation

Human peripheral blood mononuclear cells (PBMC) were separated from heparinized whole-blood by density gradient centrifugation (20 min, 20 °C, 400 × *g*) over Ficoll-Paque (Amersham, Uppsala, Sweden). Isolation of NK cells, by negative magnetic selection using the MACS system (Miltenyi Biotec GmbH, Bergisch Gladbach, Germany), has been described previously (Baumann et al., 2005). Freshly isolated NK cells were used at purity >95%. At each step, cells were counted at least twice using a Neubauer hemocytometer (VWR international, Dietikon, Switzerland) and cell viability was assessed by trypan blue exclusion test.

### Flow cytometry

The cell surface expression of a large panel of cell type-specific antigens was analyzed by flow cytometry. Pre- and post-exercise PBMC or purified NK were antibody-stained and analyzed on a FACSCanto (BD Biosciences, Franklin Lakes, NJ, USA) or Attune (Life Technologies, Basel, Switzerland). Briefly, 1 × 10^5^ cells were resuspended in staining buffer (PBS, 0.1% BSA), and incubated for 30 min at 4 °C with saturating amounts of antibody (Ab) as previously described (Forte et al., 2000). The following direct Ab were purchased from BD Biosciences: AF488-conjugated anti-CD56 (mouse IgG1), APC-conjugated anti-CD3 (mouse IgG1), FITC conjugated anti-CD16 (mouse IgG1), PE-conjugated anti-CD25 (mouse IgG1), anti-CD56 (mouse IgG1), anti-CD69 (mouse IgG1), and anti-NKG2D (mouse IgG1). From R&D Systems (Abingdon, UK): anti-CD158a (KIR2DL1)-FITC, anti-CD158b2 (KIR2DL3)-PE. From BioLegend (Luzern, Switzerland): anti-CD3-PerCPCy5.5, anti-CD158e (KIR3DL1)-FITC, anti-CD94-FITC, anti-CD282 (TLR2)-PE, anti-CD56 BrillantViolet421. From eBioscience: CD16-eFlourN605. PE-conjugated anti-NKp30 (mouse IgG1), anti-NKp44 (mouse IgG1), anti-NKp46 (mouse IgG1), and anti-DNAM-1 (mouse IgG1) were provided by Miltenyi Biotec. Irrelevant AF488-, APC-, FITC-, PE- conjugated MOPC21 antibodies (mouse IgG1; BD Biosciences) were used as isotype-matched control. Data were obtained by gating on lymphocytes in the forward scatter/side scatter plots and shown as percentages (see Figure S1 in Supplementary Material for gating strategy). To compare the levels of surface expression, the mean fluorescence intensity ratios (MFIR) were calculated by dividing the geometric mean fluorescence intensity of each sample by the geometric mean fluorescence intensity of the control Ab. For cell viability evaluation live/dead fixable Aqua stain was used according manufacturer’s specifications (Life Technologies).

### Stimulation of human NK cells

As previously reported (Millard et al., 2010; Li et al., 2013), recombinant human IL-2 (50 or 500 U/mL; Peprotech, Rocky Hill, NJ, USA), IL-12 (0.5 ng/mL; R&D Systems, Abingdon, UK), or the TLR2 agonists: lipoteichoic acid (LTA) from *S. aureus* (1 or 10 μg/mL; Sigma), and the synthetic triacylated lipopeptide Pam3CSK4 (0.5 or 5 μg/mL; Invivogen, San Diego, CA, USA) were added to NK cell cultures for the indicated periods of time.

### Single cell capture analysis of NK cell IFNγ secretion

The MACS Cytokine Secretion Assay (Miltenyi Biotec) was used to identify NK cells secreting IFNγ, according to the manufacturer’s instructions. Briefly, purified human NK cells (0.5 × 10^6^) were incubated with human IL-2/IL-12 or TLR2 agonists (LTA, Pam3CSK4) for 4 or 24 h in AIM-V medium supplemented with 2% HEPES (Invitrogen, Basel, Switzerland). Thereafter, a catching antibody (anti-IFNγ Ab conjugated to a cell surface-specific Ab) was added for 45 min during incubation at 37 °C. After washing, the cells were stained with PE-conjugated anti-IFNγ and AF488-conjugated anti-CD56 for 20 min at 4 °C. Isotype-matched Ab were used as controls. After washing, the samples were analyzed by flow cytometry.

### Measurement of IFNγ concentrations in cell culture supernatants by ELISA

Freshly isolated human NK cells (5 × 10^5^, 2.5 × 10^5^, and 1.25 × 10^5^) were incubated with recombinant human IL-2 (50 or 500 U/mL) in 96 wells in a total volume of 100 μL AIM-V medium. After 48 h, 100 μL supernatants were harvested and assayed for IFNγ secretion using an ELISA kit according to manufacturer’s instructions (Mabtech AB, Cincinnati, OH, USA).

### CD107a degranulation and intracellular IFNγ detection

NK cell degranulation was analyzed by the expression of CD107a, as previously reported (Alter et al., 2004; Millard et al., 2010). The MHC class I negative K562 cell line, was purchased from ATCC (Manassas, VA, USA). Following cytokine stimulation overnight with IL-2/IL-12 purified NK cells were incubated with K562 at an effector-to-target (E:T) ratio of 5:1 for 6 h. CD107a Ab (BD Biosciences) or MOPC21 as isotype-matched control Ab, was added directly to the cultures. After 1 h of stimulation with K562, GolgiStop reagent (2 mM, BD Biosciences) was added to the cultures and incubated for another 5 h. Cells were then stained with anti-CD56, -CD3, or isotype control Ab for 30 min at 4 °C. Next, samples were fixed, permeabilized according to manufacturer’s instructions, and stained for intracellular IFNγ for 30 min at 4 °C. The expression of surface CD107a and intracellular IFNγ was analyzed in the CD3^−^CD56^+^ NK cell fraction by flow cytometry analysis (Li et al., 2013).

### ^51^Cr-release cytotoxicity assays

The cytotoxic activity of freshly isolated or IL-2 activated human NK cells was assessed in a 4h ^51^Cr-release assay in serum-free AIM-V medium (Invitrogen, Basel, Switzerland) as previously described (Seebach et al., 1996). Briefly, ^51^Cr-labeled K562 target cells were added to triplicate samples of serial twofold dilutions of NK cells. Various E:T ratios were assessed in each experiment ranging from 40:1 to 5:1. After 4 h of incubation at 37 °C, ^51^Cr-release in the supernatants was analyzed on a gamma counter and the percentage of specific lysis was calculated.

### Statistical analysis

Data are presented as mean ± standard deviation (SD) representing experiments. Statistical analysis was performed using StatView IV software (Abacus Concepts INC., Berkeley, CA, USA). Comparisons were performed using paired two tailed Student’s *t* test and significant differences are indicated in the graph as follows: *p < 0.05, *p < 0.01, *p < 0.001.

## Results

### Brief exercise increases the absolute number and frequency of circulating NK cells

**Figure 1:**
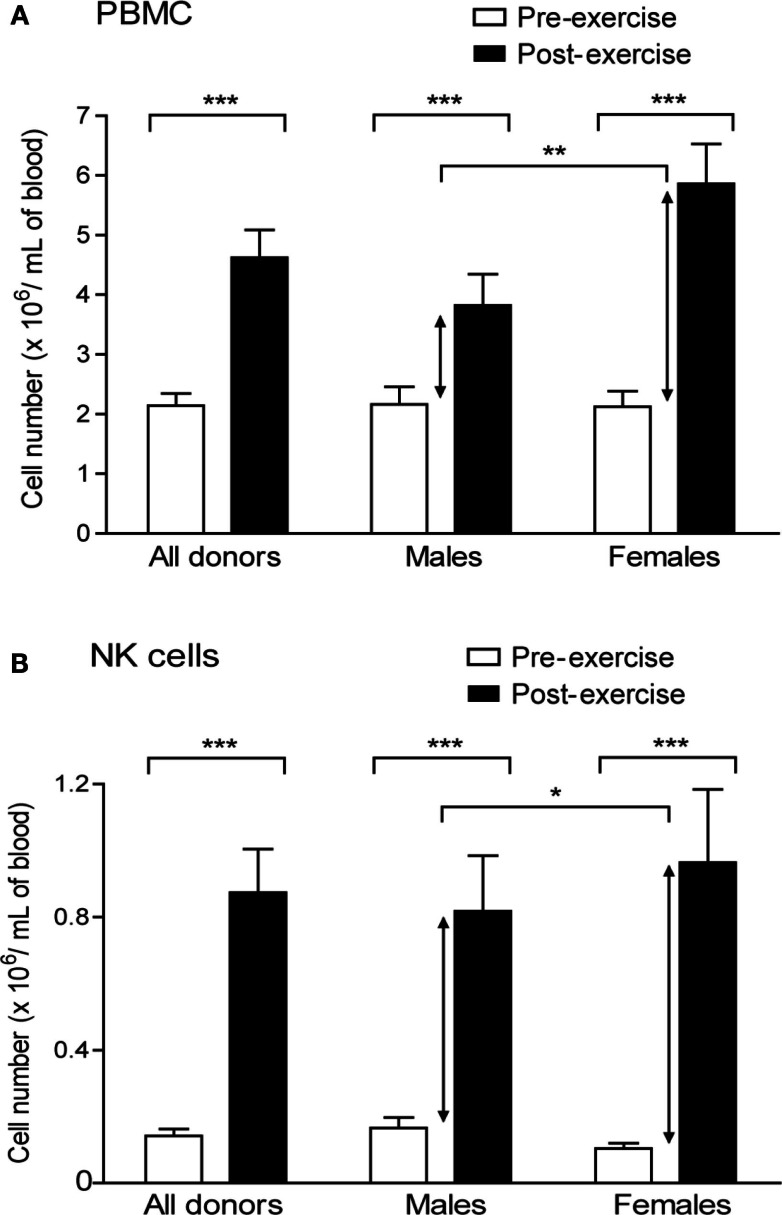
**Brief exercise increases the number and frequency of NK cells**. **(A)**. PBMC were isolated from the peripheral blood drawn either pre-exercise (white) and post-exercise (black) and extensively counted using a Neubauer hemocytometer. Shown are the mean counts (cells × 10^6^/mL ± SD) of all donors (*n* = 21), males only (*n* = 14), or females only (*n* = 7). **(B)**. NK cells were subsequently purified from PBMC and extensively counted using a Neubauer hemocytometer. Shown are the mean counts (cells × 10^6^/mL ± SD) of all donors (*n* = 21), males only (*n* = 14), or females only (*n* = 7). Significant differences are indicated in the graph as follows: *p < 0.05, **p < 0.01, ***p < 0.001 (paired two tailed student’s *t* test).

We first quantified the impact of brief exercise, consisting in running up and down of 150 stair-steps, on the number of circulating NK cells. The numbers of PBMC isolated by Ficoll gradient centrifugation from 50 mL of blood, drawn before and immediately after exercise, as well as the absolute numbers of NK cells isolated by magnetic bead selection from these PBMC were counted. PBMC numbers increased post-exercise (mean increase: 2.3-fold; *p* < 0.0001) with a greater rise in female than in male donors (Figure 1A). Corroborating previous reports, the absolute number of CD56^pos^CD3^neg^ NK cells isolated from post-exercise PBMC was significantly higher, ranging from 1.8 to 19.6-fold (*n* = 24, mean: 6.3-fold; *p* < 0.0001). As for PBMC, but to a lower extent, the rise was statistically greater in the female than in male volunteer group (mean increase: male 5.2 ± 0.8, *n* = 11; female 8.9 ± 1.4, *n* = 7; *p* = 0.022; Figure 1B).

### Effect of brief exercise on NK cell surface receptor expression

To investigate the effect of brief exercise on the phenotype of circulating NK cells, we measured the cell surface expression of several activation and inhibitory markers in total PBMC gating on total CD3^neg^CD56^pos^ NK cells, as well as on the CD56^bright^CD16^neg^/^dim^ and CD56^dim^CD16^bright^ subsets (gating strategy shown in Figure S1 in Supplementary Material). As shown in Table 1 and Figure S2 in Supplementary Material our data at baseline before running confirm the previously published differences between the CD56^bright^ and CD56^dim^ NK cell subsets, i.e., higher expression of CD25, NKG2D, CD94/NKG2A/C, and lower expression of KIR and CD16 on the CD56^bright^ subset. Accordingly, no statistically significant differences were observed for CD69, NCR, DNAM-1, and TLR2 between the CD56^bright^ and CD56^dim^ NK cell subsets. Following brief exercise, the percentage of CD56^bright^CD16^neg^/^dim^ in the total CD56^pos^CD3^neg^ NK cell population decreased significantly (see also Figure 6), as well as the percentages of CD94, NKG2A, and DNAM-1. It is of note that DNAM-1 expression decreased equally in both NK cell subsets. The slight decrease of CD94/NKG2A in the total NK cell population can be explained by the decrease of the highly CD94/NKG2A positive CD56^bright^ subset associated by a slight decrease of CD94 expression itself on the CD56^dim^ subset. As expected, the expression of KIR2DL1, KIR2DL3, and KIR3DL1, which was measured by mAb specific for the long-tailed inhibitory isoforms, turned out to be higher on CD56^dim^ NK cells, but did not change significantly following running. Finally, NKG2D and NCR expression were not affected by exercise with the exception of a tendency to decrease for NKp44 expression. Being aware of the difficulty to clearly identify the percentage of positive cells which depends on correctly setting the cut-off defined by the isotype control antibody staining in the case of overlapping FACS histograms (Figure S2 in Supplementary Material), we also analyzed the levels of expression by MFIRs. However, this analysis did not add any relevant additional information and is thus not shown.

**Table 1:** **The effect of brief exercise on the expression of NK cell surface markers (%)**.

Marker	All CD56^pos^ NK cells		CD56^bright^ NK cells		CD56^dim^ NK cells	
	Pre-exercise	Post-exercise	Pre-exercise	Post-exercise	Pre-exercise	Post-exercise
CD56^+^CD3^–^			6.74 ± 5.77	3.45 ± 3.02*	89.67 ± 7.81	94.28 ± 4.08*
CD69^+^	7.58 ± 7.89	1.28 ± 0.60	5.63 ± 5.12	7.08 ± 4.25	4.43 ± 6.11	1.25 ± 0.63
CD25^+^	0.21 ± 0.20	0.25 ± 0.19	1.92 ± 3.1	1.62 ± 2.10	0.13 ± 0.16	0.19 ± 0.15
NKG2D^+^	29.71 ± 18.38	31.02 ± 6.63	42.29 ± 25.56	54.30 ± 9.29	24.81 ± 21.44	31.70 ± 7.03
CD94^+^	65.54 ± 7.42	59.79 ± 7.47^***^	96.65 ± 2.91	94.52 ± 4.74	61.57 ± 6.73	57.31 ± 7.56^***^
NKG2C^+^	32.39 ± 15.95	32.26 ± 15.32	82.73 ± 14.79	82.11 ± 11.27	29.36 ± 15.68	30.09 ± 15.07
NKp30^+^	34.82 ± 15.89	39.46 ± 14.39	40.18 ± 17.09	49.08 ± 16.07	37.41 ± 16.07	40.06 ± 14.94
NKp44^+^	0.32 ± 0.44	0.12 ± 0.17	1.18 ± 1.58	0.48 ± 1.08^*^	0.36 ± 0.58	0.12 ± 0.17
NKp46^+^	70.56 ± 18.46	79.53 ± 5.21	84.15 ± 29.91	98.20 ± 1.03	72.09 ± 15.90	79.09 ± 6.66
DNAM-1^+^	33.12 ± 10.80	15.78 ± 8.26^*^	30.60 ± 11.15	15.95 ± 9.11^**^	37.26 ± 12.34	16.81 ± 8.88^*^
KIR2DL1^+^	34.42 ± 11.53	36.42 ± 11.12	19.90 ± 10.92	19.42 ± 5.60	35.08 ± 11.03	36.30 ± 11.06
KIR2DL3^+^	31.31 ± 13.11	33.47 ± 10.27	21.20 ± 13.67	17.99 ± 5.38	32.78 ± 12.94	34.49 ± 10.20
KIR3DL1^+^	19.69 ± 10.34	21.58 ± 10.20	9.80 ± 5.29	12.22 ± 5.98	20.18 ± 10.67	21.42 ± 10.54
NKG2A^+^	48.28 ± 7.04	45.79 ± 7.28^**^	80.73 ± 11.69	81.71 ± 13.66	45.92 ± 7.34	44.74 ± 7.30
TLR2^+^	23.62 ± 9.20	23.62 ± 8.88	24.73 ± 8.13	16.87 ± 8.13	24.11 ± 9.33	24.19 ± 9.11

The cell surface expression of a large panel of NK cell associated antigens was analyzed on PBMC stemming from at least five different donors by flow cytometry. Data were obtained by gating on single living lymphocytes in the forward scatter/side scatter plots and shown as mean percentages ± SD. Significant differences are indicated in the graph as follows: *p < 0.05, **p < 0.01, ***p < 0.001 (paired two tailed student’s *t* test).

### Brief exercise does not interfere with IFNγ secretion or NK cytotoxicity but slightly decreases NK cell degranulation

**Figure 2:**
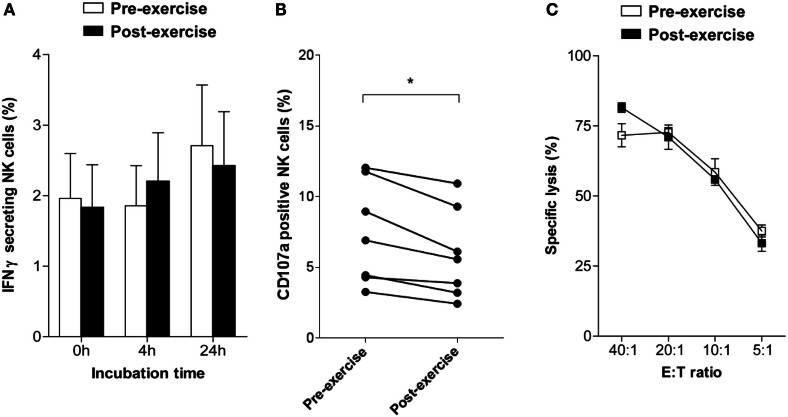
**Changes in NK cell activity following brief exercise**. **(A)** Pre-exercise (white) and post-exercise (black) isolated NK cells were analyzed by flow cytometry to determine the percentage of IFNγ-secreting cells using an IFNγ capture assay, either immediately upon isolation or following culture in AIM-V medium supplemented with 2% HEPES without cytokines for 4 or 24 h. Data represent the mean% ± SD of seven different donors. **(B)** The frequency of CD107a expressing NK cells was assessed by flow cytometry immediately after isolation in response to incubation for 6 h with K562 cells without cytokine stimulation. Using a forward scatter/side scatter lymphocyte gate anti-CD107a and anti-CD56 Ab double positive NK cells were determined. Shown is the percentage of CD107a-positive NK cells of six different donors. Significant differences are indicated as follows: *p < 0.05 (paired two tailed student’s *t* test), **(C)** Cytotoxicity of pre-exercise (white) and post-exercise (black) NK cells against K562 target cells was analyzed immediately after isolation using ^51^Cr-release assays. Cytotoxicity was determined and expressed as percent specific lysis at different E:T ratio. Data represent the means ± SD of triplicates and are representative for one out of three donors.

To evaluate whether brief exercise interferes with NK cell function three parameters were analyzed directly following isolation in the absence of exogenous stimulation: the frequency of IFNγ-secreting NK cells using an IFNγ capture assay, NK cell degranulation by staining for CD107a, and NK cytotoxicity by ^51^Cr-release assays. Before exercise, the frequency of IFNγ-secreting NK cells at baseline was low ranging from 0.3 to 5.3% (mean: 2.0 ± 1.7; *n* = 7). Exercise had no direct effect on the capability of NK cells to secrete IFNγ, neither immediately upon isolation nor following 4 or 24 h *in vitro* culture in AIM-V medium supplemented with 2% HEPES (Figure 2A). The frequency of CD107a-positive NK cells was low and slightly decreased after brief exercise from 7.38 ± 3.62 to 5.91 ± 3.17% (*p* = 0.002) (Figure 2B). Finally, spontaneous NK cell cytotoxicity as evaluated by ^51^Cr-release assays using NK-sensitive K562 target cells was similar pre- and post-exercise in all 5 donors tested (Figure 2C).

### Cytokine-induced IFNγ production and secretion by NK cells isolated following brief exercise

**Figure 3:**
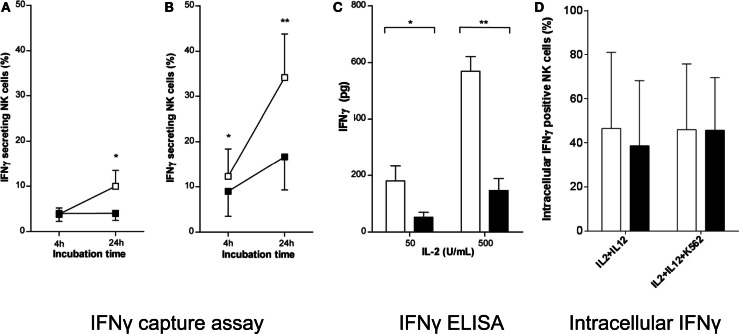
**IL-2 does not fully induce IFNγ secretion by NK cells isolated following brief exercise**. Pre-exercise (white) and post-exercise (black) isolated NK cells were treated with **(A)** 50 U/mL or **(B)** 500 U/mL IL-2. After 4 and 24 h treatment the percentage of IFNγ-secreting NK cells was determined by flow cytometry using an IFNγ capture assay. Data shown represent the mean% ± SD of seven different donors. **(C)** After 48 h of IL-2 treatment, the amount of IFNγ produced by NK cell cultures (1 × 10^5^ cells/well) was measured by ELISA. Data represent the mean pg/culture ± SD of five independent experiments with cells from different donors. Significant differences are indicated in the graphs as follows: *p < 0.05, **p < 0.01 (paired two tailed student’s *t* test). **(D)** Pre-exercise (white) and post-exercise (black) isolated intracellular IFNγ positive NK cells after overnight incubation with 50 U/mL of IL-2 and 0.5 ng/mL of IL-12 followed by additional 6 h of incubation with or without K562 cells at a NK to K562ratio of 5:1 in the presence of GolgiStop. Data represent the mean% ± SD of 5 independent experiments with cells from different donors.

We then evaluated whether brief exercise may interfere with the NK cell response to cytokine stimulation *in vitro*. As previously reported, IL-2 stimulation induced low levels of IFNγ secretion by purified human peripheral blood NK cells in short-term cultures (Krishnaraj and Bhooma, 1996), whereas combined IL-2/IL-12 stimulation was a much more powerful trigger of IFNγ secretion (Li et al., 2013). Here, NK cells isolated pre- and post-exercise, respectively, were treated with either low- (50 U/mL) or high- (500 U/mL) doses of IL-2. The percentage of IFNγ-secreting NK cells was determined 4 and 24 h later using the IFNγ-capture assay, and the overall production of IFNγ per culture was measured by ELISA after 48 h. Stimulation of NK cells isolated pre-exercise with IL-2 (low or high dose), led to a significant increase in the frequency of IFNγ-secreting cells at both time-points tested. On the contrary, when NK cells were isolated post-exercise IL-2 stimulation failed to increase the frequency of IFNγ-secreting cells (Figures 3A,B). When the total amount of IFNγ secreted into the NK culture supernatants was evaluated upon stimulation with IL-2 (low and high dose), pre-exercise isolated NK cells rapidly produced large amounts of IFNγ, whereas a significantly reduced production was observed using NK cells isolated post-exercise (Figure 3C). Finally, to test whether brief exercise had an effect on the capacity of NK cells to respond to strong cytokine stimuli, purified NK cells were cultured overnight in the presence of both IL-2 (50 U/mL) and IL-12 (0.5 ng/mL) with and without an additional stimulation by K562 cells for 6 h and analyzed for intracellular IFNγ production. Baseline intracellular IFNγ production was below 1% of NK cells before cytokine stimulation (data not shown) and significantly increased following IL-2/IL-12 stimulation (40–50% of NK cells), whereas K562 stimulation had no additional effect. Importantly, there was no significant difference in intracellular IFNγ production before and after exercise (Figure 3D), and no significant difference when CD56^bright^ and CD56^dim^ NK cells were analyzed separately (Figure S3B in Supplementary Material).

### Degranulation and cytotoxicity of NK cells isolated following exercise in response to cytokine and target cell stimulation

**Figure 4:**
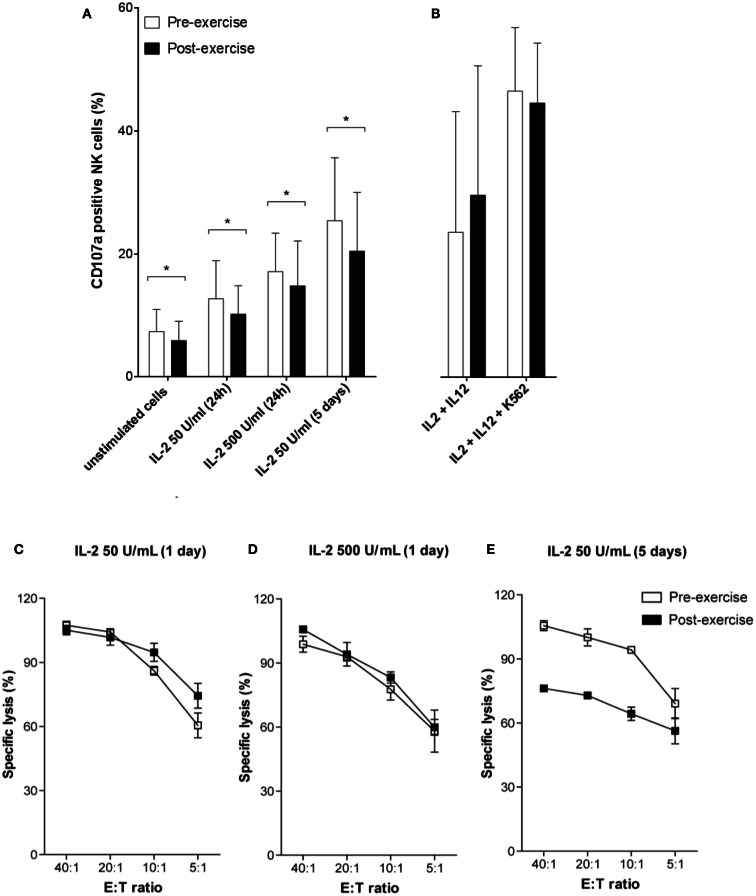
**NK cell degranulation and cytotoxicity in response to IL-2/IL-12 treatment**. **(A)** The percentage of degranulation in purified NK cells was assessed pre-exercise (white) and post-exercise (black) by flow cytometry using anti-CD107a and anti-CD56 Ab staining. Following treatment with 50 or 500 U/mL IL-2 for 24 h or 5 days, NK cells were incubated for additional 6 h with K562 target cells. **(B)** Stimulation of purified NK cells by combined overnight incubation with 50 U/mL of IL-2 and 0.5 ng/mL of IL-12 followed by additional 6 h of incubation with or without K562 cells. Data were obtained by gating on a forward scatter/side scatter lymphocyte gate to exclude K562 cells. Shown are the mean percentages ± SD of degranulating CD107a^+^ NK cells for at least five different donors. Significant differences are indicated in the graphs as follows: *p < 0.05 (paired two tailed student’s *t* test). Cytotoxicity of pre-exercise (white) and post-exercise (black) NK cells treated with IL-2 **(C)** 50 U/mL for 24 h, **(D)** 500 U/mL for 24 h, and **(E)** 50 U/mL for 5 days, was analyzed in ^51^Cr-release assays using K562 target cells. Data are shown as percent specific lysis at different E:T ratios and represent the means ± SD of triplicates. Results shown are all from one donor and representative for the results of three different donors.

To analyze whether brief exercise interferes with IL-2/IL-12 and K562-stimulated NK cell degranulation, we quantified CD107a expression on both pre- and post-exercise isolated NK cells. IL-2 treatment dose-dependently and time-dependently increased degranulation of both pre- and post-exercise isolated NK cells. However, NK cells isolated following exercise consistently exhibited a slightly reduced CD107a degranulation capacity compared to pre-exercise NK cells, and all conditions of IL-2 stimulation tested failed to restore comparable amounts of CD107a degranulation (Figure 4A). Combined stimulation with IL-2/IL-12, and even more with additional K562 stimulation, induced higher levels of CD107a expression without significant differences between NK cells isolated before and after brief exercise (Figure 4B), and without significant differences between CD56^bright^ and CD56^dim^ NK cells (Figure S3A in Supplementary Material). In a second set of experiments, the impact of brief exercise on ^51^Cr-release NK cytotoxicity in response to IL-2 stimulation was evaluated. After 24 h with low or high dose of IL-2 treatment, NK cells isolated pre- and post-exercise both retained comparable cytolytic activity (Figures 4C,D). However, after 5 days of culture with IL-2, NK cells isolated pre-exercise exhibited enhanced cytotoxicity as compared to post-exercise NK cells (Figure 4E).

### TLR2 agonist treatment fails to induce IFNγ production by NK cells isolated following brief exercise

**Figure 5:**
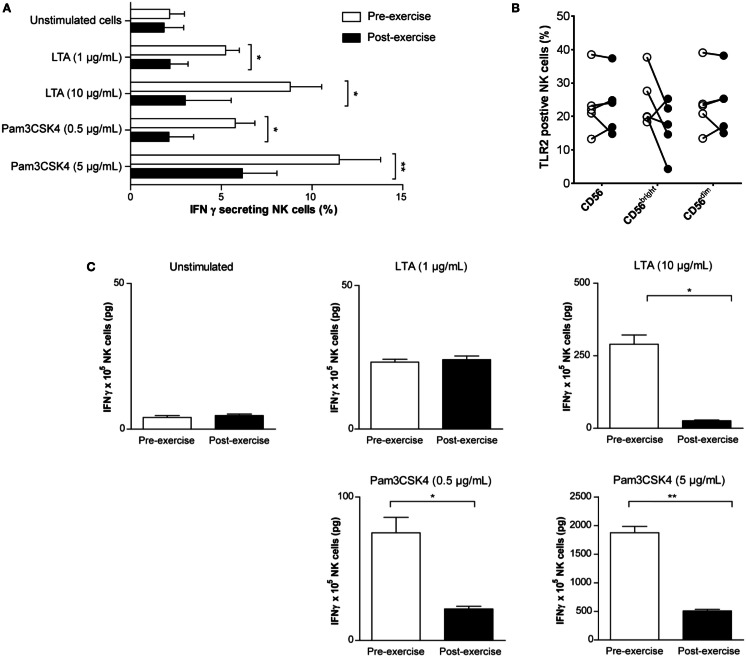
**TLR2 agonists do not fully induce IFNγ production by NK cells isolated following brief exercise**. Pre-exercise (white) and post-exercise (black) isolated NK cells were treated with TLR2 agonists. **(A)** NK cells were either left untreated or stimulated with LTA (1 or 10 μg/mL) or Pam3CSK4 (0.5 or 5 μg/mL). After 24 h, the frequency of IFNγ producing NK cells was determined by flow cytometry using an IFNγ capture assay. Data represent the mean% ± SD of five different donors. **(B)** Surface expression of TLR2 on total CD56^pos^/CD3^neg^ NK cells, CD56^bright^, and CD56^dim^ subsets measured before and after exercise. Data were obtained analyzing PBMC by gating on living cells in a forward scatter/side scatter lymphocyte gate; followed by gating on CD56^pos^/CD3^neg^ NK cells, CD56^bright^ and CD56^dim^ NK cells were identified based on CD56 and CD16 expression (see Figure S1 in Supplementary Material). Shown are the mean% ± SD of five different donors. **(C)**, Total IFNγ (pg) produced by NK cell cultures (1 × 10^5^ cells/well) was measured by ELISA after 48 h TLR2 agonist treatment and compared to unstimulated cultures. Shown are the means (pg) ± SD of triplicates and the results are representative for one out of three donors. Significant differences are indicated in the graph as follows: *p < 0.05, **p < 0.01 (paired two tailed student’s *t* test).

Since several recent studies emphasized the importance of TLR expression and TLR-mediated activation in NK cell immunobiology (Della et al., 2005) we next investigated whether brief exercise also impairs TLR agonist-driven *in vitro* activation of NK cell functions. In a previous study, we had demonstrated direct activation of human NK cells by stimulation with TLR2 agonists (LTA and Pam3CSK4), but not with TLR4 agonists (Millard et al., 2010). Treatment of pre-exercise NK cells with LTA and Pam3CSK4 increased the frequency of IFNγ-secreting cells using a capture assay (Figure 5A). A reduced effect for both TLR agonists was observed when post-exercise isolated NK cells were stimulated. Because this result theoretically could be explained by differences in TLR2 expression, the latter was analyzed by flow cytometry (Table 1; Figure 5B). While there was no apparent difference in the total CD56^pos^ NK population, TLR2 expression was slightly reduced in the CD56^bright^ NK subset following running. However, this finding did not reach statistical significance (24.7% ± 8.1 and 16.9% ± 8.1, pre- and post-exercise, respectively, *p* = 0.13, *n* = 5). In line with the lower number of IFNγ-secreting cells (Figure 5A), and the lower expression of TLR2 on CD56^bright^ NK cells (Figure 5B), LTA and Pam3CSK4 dose-dependently up-regulated the amount of secreted IFNγ in the supernatants of pre-exercise NK cell cultures (Figure 5C). Whereas LTA 100 μg/ml failed to increase IFNγ secretion in post-exercise NK cells, only a slight up-regulation was observed with the highest dose of Pam3CSK4. Thus, activation of NK cells in response to stimulation for 24 or 48 h with TLR2 agonists *in vitro* was significantly reduced when the cells were isolated after running.

**Figure 6:**
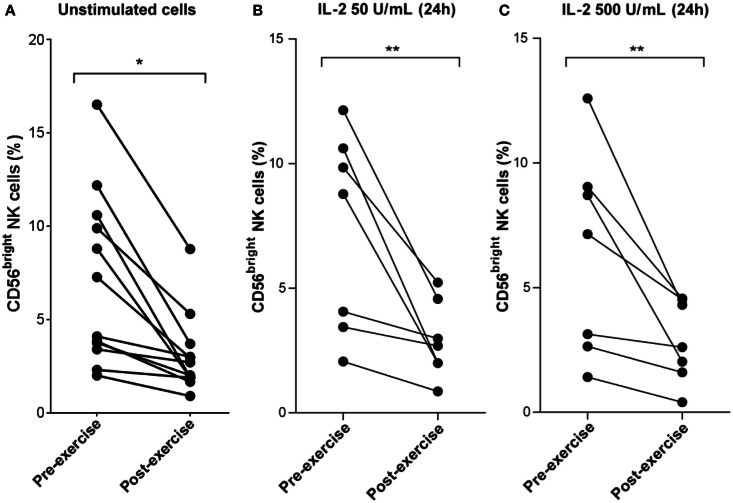
**Decrease of CD56^bright^ NK cell frequency following brief exercise is not affected by 24 h of *in vitro* IL-2 treatment**. **(A)** Pre-exercise and post-exercise NK cells (*n* = 7) or PBMC (*n* = 5) were isolated and stained immediately with a mix of anti-CD3/anti-CD56 Ab or anti-CD3/anti-CD56/anti-CD16, respectively. The frequency of CD56^dim^ versus CD56^bright^ NK cells was then quantified by flow cytometry. Shown are the mean percentages of CD56^bright^ NK cells detected in 12 different donors. **(B)** Pre-exercise and post-exercise NK cells were isolated and stimulated with 50 U/mL IL-2 or **(C)** 500 U/mL IL-2 for 24 h. The frequency of CD56^bright^ NK cells of seven different individuals pre- and post-exercise was determined. Significant differences are indicated in the graphs as follows: **p < 0.01 (paired two tailed student’s *t* test).

### Brief exercise decreases the frequency of CD56^bright^ NK cells

Finally, the effect of brief exercise on NK cell subsets with respect to CD56 expression was analyzed (see Figure S1 in Supplementary Material for the gating strategy). As illustrated in Figure 6A and Table 1, the frequency of CD3^-^CD56^bright^ NK cells was significantly reduced post-exercise (mean decrease: 2.4-fold, pre: 7.1% ± 4.6, post: 3.1% ± 2.1, *n* = 12, *p* = 0.012) in all donors tested. *In vitro* NK cell stimulation with low (50 U/mL; Figure 6B) or high (500 U/mL; Figure 6C) doses of IL-2 did not affect the exercise-induced decrease in the CD56^bright^ NK cell fraction (mean decrease for low-dose IL-2: 2.4-fold, *p* = 0.021 and mean decrease for high dose IL-2: 2.5-fold, *p* = 0.03). Finally, stimulation with TLR2 agonists also failed to reverse the exercise-induced decrease in the CD56^bright^/CD56^dim^ NK cell ratio (data not shown).

## Discussion

NK cells not only exert natural cytotoxicity against virus infected or neoplastic cells, but they are also potent producers of immunoregulatory cytokines. A new understanding of the regulatory functions of NK cells, e.g., in the context of solid organ transplantation or during inflammation, has recently emerged (Pratschke et al., 2008; Vivier et al., 2011). However, several aspects of NK cell biology including the fact that physical exercise leads to a significant increase of the number of circulating NK cells remain to be unveiled (Walsh et al., 2011). Here, we investigated whether and how a single bout of brief exercise, consisting in running up and down 150 stair-steps, had an impact on NK cell phenotype and function.

Earlier reports have demonstrated that exercise of various types, durations, and intensities are able to induce NK cell recruitment to the blood stream (Pedersen and Hoffman-Goetz, 2000). Here, we show that the number of NK cells can be multiplied more than sixfold simply by running some flights of stairs without significant functional changes immediately after isolation. Remarkably, the exercise-induced increases in PBMC and NK cell numbers were significantly higher in females than in males. Indeed, gender-associated differences in immunological responses have been previously reported. Girls showed a 35% greater increase of NK cell counts than boys of similar pubertal status following 60 min of cycling (Timmons et al., 2006). Recent studies also demonstrated that a brief bout of exercise alters gene expression and distinct gene pathways including pathways linked to NK cytotoxicity in PBMC of boys (Radom-Aizik et al., 2009b) and even to a higher extent of early- and late-pubertal females (Radom-Aizik et al., 2009a). Thus, exercise-induced NK cell recruitment to the circulation can be added to a variety of physiological responses to exercise that are evidently under the influence of sex hormones such as muscle fatigue, temperature regulation, or endocrine responses.

Exercise mobilizes NK cells within only a few minutes from the marginal pool into the blood stream, presumably via increased shear stress and spleen-independent β2-adrenergic mechanisms (Pedersen and Hoffman-Goetz, 2000). Parenthetically, similar results were observed in response to psychological stress (Bosch et al., 2005). Moreover, a selective mobilization of cytotoxic CD56^dim^ NK cells by epinephrine has been demonstrated (Dimitrov et al., 2010). A decrease in the CD56^bright^/CD56^dim^ NK cell ratio was also reported in children during exercise, followed by an increase at 30 min of recovery (Timmons et al., 2006). These data suggest that the epinephrine-triggered detachment affects CD56^dim^ NK cells to a higher extent, probably due to a higher expression and/or higher affinity of β2-adrenergic receptors (Molenaar et al., 2007). During exercise, the plasma concentration of epinephrine and norepinephrine increases and it has been postulated that catecholamines differentially modulate the expression of adhesion molecules on NK cells (Shephard, 2003). Indeed, the expression of CD44 and CD18 was reduced on human NK cells during exercise and *in vitro* β2-adrenergic stimulation caused detachment of NK cells from cultured endothelial cells (Benschop et al., 1993). Our results showing a 6-fold average increase of CD56^dim^ versus an only 2.5-fold increase of CD56^bright^ NK cells are hence in line with previous reports showing that CD56^bright^ NK cells are less responsive to immediate stress-induced mobilization than their CD56^dim^ counterparts (Campbell et al., 2009). CD56^bright^ NK cells expressed higher levels of the inhibitory heterodimeric receptor CD94/NKG2A/C, NKG2D, the high-affinity IL-2 receptor (CD25); lower levels of KIR and CD16; and there were no major differences for TLR2, DNAM-1; NCR, and CD69 expression. Taken together, our phenotype analysis revealed changes on NK cells isolated after brief exercise that mainly can be explained by the higher frequency of CD56^dim^ NK cells. The only marker that decreased in both NK cell subsets after running was DNAX accessory molecule-1 (DNAM-1) which plays a role in tumor immunosurveillance (Morgado et al., 2011). Stimulation of DNAM-1 by its ligands CD155 (poliovirus receptor) and CD112 (nectin-2) expressed on different types of tumors including melanoma, leukemia, and ovarian carcinoma leads to NK cell activation and target cell lysis.

Exercise-induced increase in NK cytotoxicity is largely due to an increase in the absolute numbers and percentages of circulating blood NK cells, whereas cytotoxicity expressed on a per cell basis does not appear to change much after acute exercise (Walsh et al., 2011). However, during and after 1 month of high-intensity competitive training both total NK cytotoxicity and lytic units per NK cell decreased whereas the circulating numbers of CD56^dim^ NK cells remained unchanged (Suzui et al., 2004). In the present study, the overall per cell NK cytotoxicity was not altered by brief exercise, the target cell line K562 was killed to the same extent by NK cells isolated before and after running. However, there was a slight but statistically significant decrease in the level of CD107a expression after running, pre 7.4% versus post 5.9% at baseline, which persisted upon IL-2 stimulation. This change cannot be attributed to the distributional shift toward CD56^dim^ following running since CD56^dim^ NK cells rather express higher levels of CD107a than CD56^bright^ NK cells (Penack et al., 2005). Potential mechanisms of downregulation include shedding or internalization induced by the β2-adrenergic triggering. On the other hand, CD107a expression on NK cells stimulated by a combination of IL-2/IL-12 and K562 cells was not significantly different before and after exercise, in both the CD56^dim^ and CD56^bright^ NK cell subsets. Intriguingly, we observed a decrease in NK cytotoxicity against K562 target cells following 5 days of IL-2 stimulation in NK cells isolated after running. This result might indicate that these NK cells are less sensitive to IL-2, either due to the known distributional shifts in NK cell subsets or by altered NK cell function on a per cell basis. Although we can only speculate, the decrease in the CD56^bright^ NK cell frequency following brief exercise may, at least partially, explain the hypo-responsiveness of post-exercise NK cells to IL-2 stimulation. In conclusion, to our knowledge this is the first study demonstrating that NK cells mobilized into the circulation in response to exercise have altered sensitivity to IL-2 stimulation.

Production and secretion of IFNγ by NK cells play an important role in both humoral and cellular immune responses. However, whether plasma levels of IFNγ change or not following exercise remains a matter of debate. Several studies reported no changes (for review see Shephard et al., 1994), whereas plasma levels of IFNα, β, and γ were increased following a bout of submaximal exercise (Viti et al., 1985). On the contrary, heavy resistance exercise (Bush et al., 2000) or 30 min bicycle exercise (Kimura et al., 2001) led to decreased IFNγ plasma concentrations. As to the effect of exercise on IFNγ production and secretion on a cellular level only limited information is available. Moderate exercise led to an increase of IFNγ production by PHA-stimulated mononuclear cells (Baum et al., 1997). On the contrary, 30 min of exhaustive exercise reduced the IFNγ level in the supernatants of whole-blood stimulated by staphylococcal enterotoxin B (SEB) significantly (Baum et al., 1997) and 60 min of bicycle exercise had no influence on IFNγ production by LPS-stimulated PBMC (Haahr et al., 1991). However, in the latter studies the use of unspecific stimuli such as LPS, SEB, and PHA as well as the heterogeneous cell populations make the identification of the cellular cytokine source of IFNγ difficult. In fact, IFNγ production in response to LPS cannot be attributed to NK cells since our group and others have demonstrated that despite the expression of TLR4 human NK cells cannot be activated by LPS (O’Connor et al., 2006; Millard et al., 2010). Therefore, the effect of brief exercise on IFNγ production and secretion by human NK cells at an individual cell level remained to be investigated. Here, we demonstrate that brief exercise had no direct effect on the low baseline level of IFNγ secretion by NK cells, but was associated with reduced IFNγ production following *in vitro* stimulation with IL-2 or TLR2 agonists. In contrast, intracellular IFNγ production in NK cells stimulated by a combination of IL-2/IL-12 in the absence or not of K562 cells was not significantly different before and after exercise. As for degranulation and cytotoxicity discussed above, the observed effect of brief exercise on the sensitivity of NK cells to IL-2 or TLR2 stimulation *in vitro* might be related to distributional changes in the population or due to modifications of the properties of existing populations. In contrast to the changes in degranulation and cytotoxicity, the decreased levels of IFNγ production are more easily explained by the decrease of the CD56^bright^ NK cells after running, since this subset is primarily responsible for the IFNγ production. The phenotype analysis of CD25 did not help us to better understand the reduced response to IL-2, since we did not observe major changes. However, brief exercise seemed to reduce the expression of DNAM-1 on all NK cells and of TLR2 on CD56^bright^ cells by unknown mechanisms. Interestingly, in another report the TLR2 ligand Pam3CSK4 failed to elicit a response on platelets that had been primed with epinephrine (Ward et al., 2005) suggesting that β2-adrenergic receptor-mediated effects on NK cells during brief exercise might be responsible for the observed reduced sensitivity of post-exercise NK cells to TLR2 agonist stimulation.

In conclusion, brief exercise increased circulating CD56^dim^ cytotoxic NK cells without major immediate functional changes, whereas subsequent NK cell activation by IL-2- and TLR2 agonist-driven stimulation were significantly impaired. Because of this potential bias, investigations of NK cell biology using exercise-induced NK cells, which otherwise can be regarded as a useful and simple tool to increase the number of cells available for *in vitro* experiments and NK cell harvesting for cell therapies for overcoming tumors, should be carefully analyzed. On the other hand, the biological and clinical relevance of exercise-induced NK cell recruitment to the circulation remains largely elusive. Presently, we can only speculate on how exercise-induced CD56^dim^ NK cells might physiologically contribute to tissue repair and/or whether these cells play a role in host defense against infections. Therefore, future studies need to address the exact mechanisms leading to the selective demargination of cytotoxic NK cells. Even more importantly, the destination(s) of mobilized NK cells following exercise-induction should be examined. A better understanding of exercise-associated NK cell trafficking and the elucidation of the exact physiological role of this impressive phenomenon are warranted.

## Conflict of Interest Statement

The authors declare that the research was conducted in the absence of any commercial or financial relationships that could be construed as a potential conflict of interest.

## Supplementary Material

The Supplementary Material for this article can be found online at http://www.frontiersin.org/NK_Cell_Biology/10.3389/fimmu.2013.00125/abstract

Figure S1 **Gating strategy to quantify the frequency of CD56^bright^ NK cells**. Pre-exercise and post-exercise PBMC were isolated, stained with live/dead fixable Aqua stain, with either the isotype controls, or anti-CD3/anti-CD56/anti-CD16 Ab followed by quantification by flow cytometry. Single living lymphocytes were gated and NK cells were defined by the expression of CD3–CD56+. Finally, the CD56^bright^ subset was distinguished from the CD56^dim^ subset with the help of CD16 expression. Shown is the gating strategy of one representative donor out of 6.

Figure S2 **Flow cytometry histograms of NK marker analysis**. Representative multicolor flow cytometry experiment depicting the histograms of several NK cell surface markers as indicated. PBMC were isolated before and after running and stained with fluorescent mAb. Single living lymphocytes were gated and CD3–CD56+ NK cells were analyzed. The expression in pre-exercise (blue line) and post-exercise (red line) NK cells is shown with their respective isotype controls (grey and black for pre- and post-exercise, respectively). The same analysis was performed to identify the expression of the NK cell marker by gating on either the CD56^bright^ or CD56^dim^ subset with the help of CD16 expression as shown in Figure S1 (data not shown).

Figure S3 **Functional analysis of CD56^bright^ and CD56^dim^ NK cells**. NK cells from five different donors were purified and stimulated overnight with IL-2 and IL-12. Following the addition or not of K652 cells as described in the M&M section degranulation of CD56^total^, CD56^bright^, and CD56^dim^ NK cells is shown as percentage of positive cells for CD107a **(A)**, whereas the percentages of positive intracellular staining for IFNγ for CD56^total^, CD56^bright^, and CD56^dim^ NK cells is shown in **(B)**. No significant differences were observed.
