# Supplementary figures and images for: Brief Exercise Increases Peripheral Blood NK Cell Counts without Immediate Functional Changes, but Impairs their Responses to ex vivo Stimulation
Source: Front Immunol. 2013 May 29;4:125. doi: 10.3389/fimmu.2013.00125 (PMC3665937; doi:10.3389/fimmu.2013.00125)

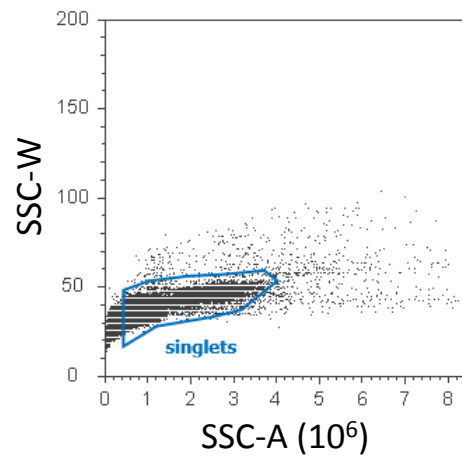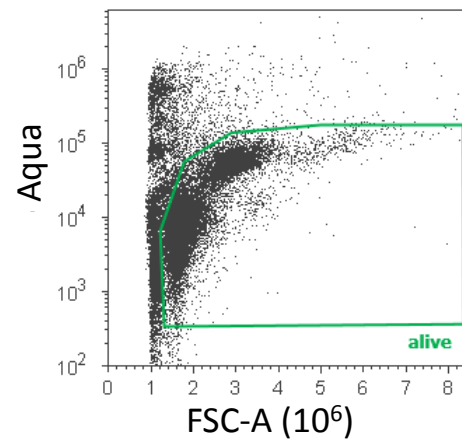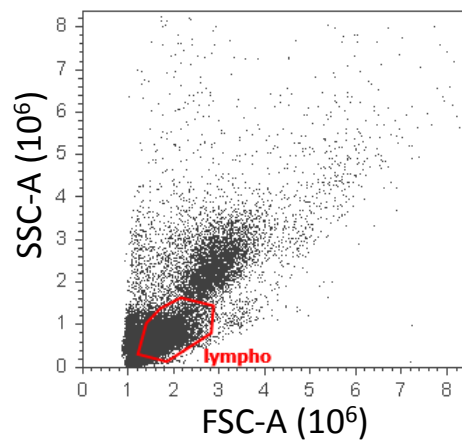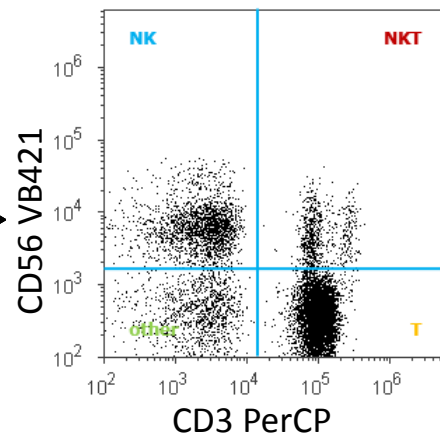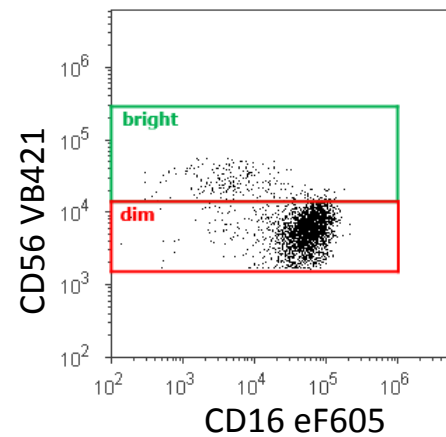

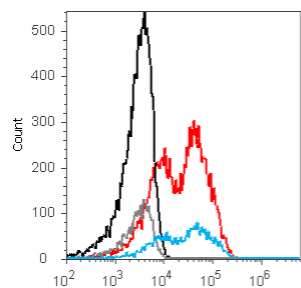

**CD94**

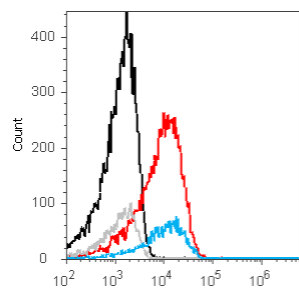

**NKG2A**

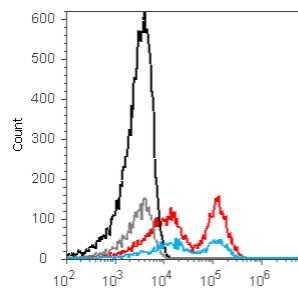

**KIR2DL1**

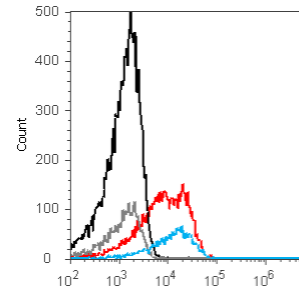

**KIR2DL3**

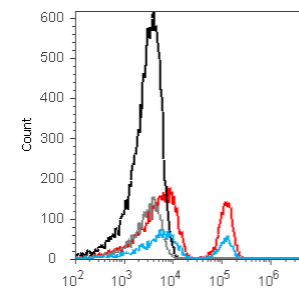

**KIR3DL1**

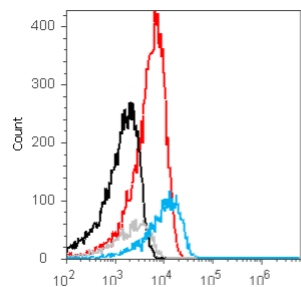

**NKG2D**

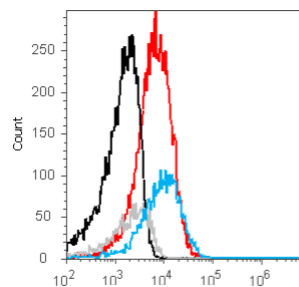

**NKp30**

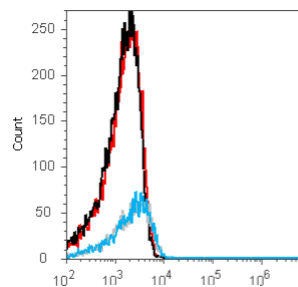

**NKp44**

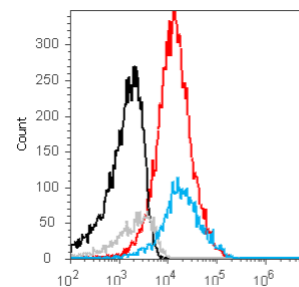

**NKp46**

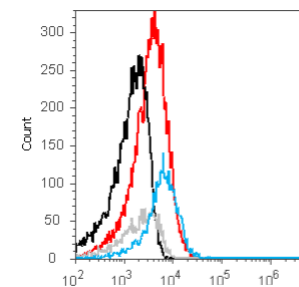

**DNAM-1**

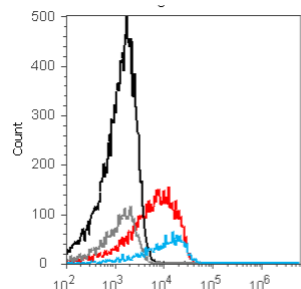

**TLR2**

A

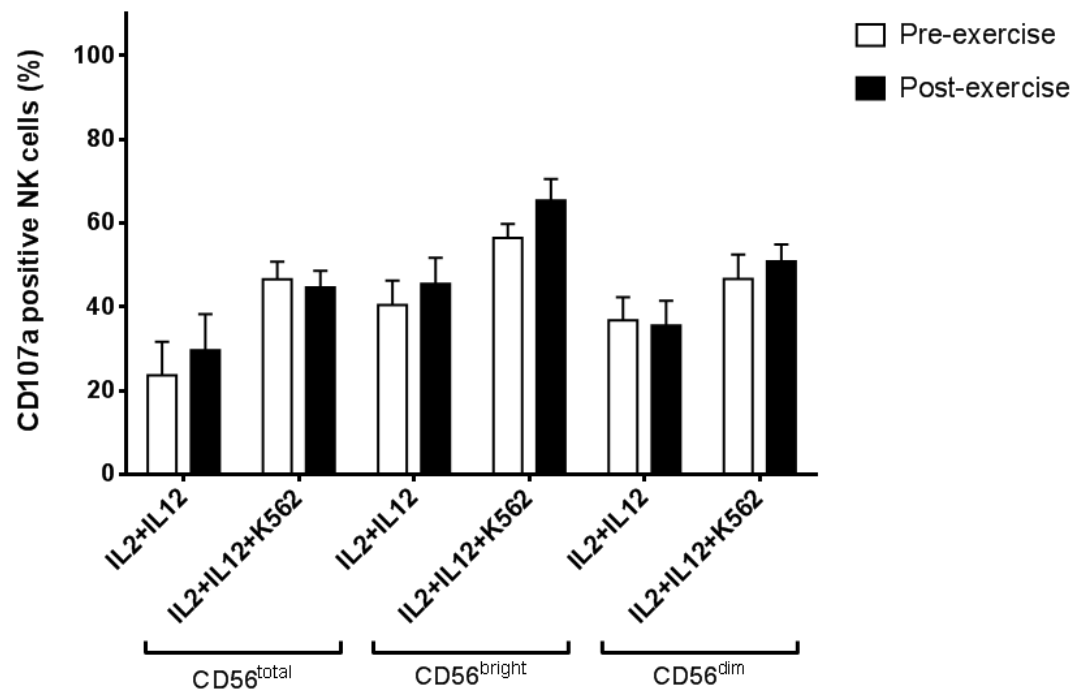

B

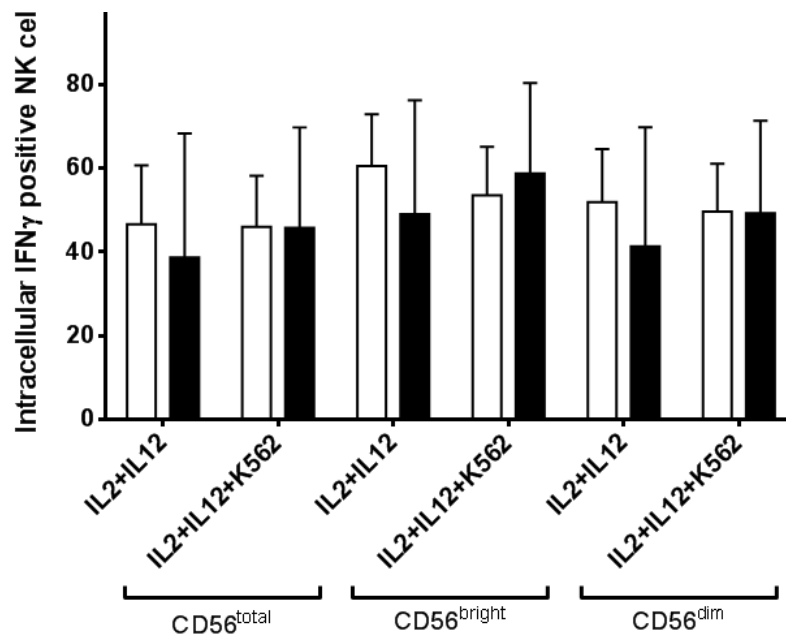

Supplement: Figure S1 — Gating strategy to quantify the frequency of CD56bright NK cells. Pre-exercise and post-exercise PBMC were isolated, stained with live/dead fixable Aqua stain, with either the isotype controls, or anti-CD3/anti-CD56/anti-CD16 Ab followed by quantification by flow cytometry. Single living lymphocytes were gated and NK cells were defined by the expression of CD3–CD56+. Finally, the CD56bright subset was distinguished from the CD56dim subset with the help of CD16 expression. Shown is the gating strategy of one representative donor out of 6. [file 39740_Seebach_Presentation1.PDF]
